# Challenges and expectations on the use of automated home cage monitoring for advancing laboratory animal care and welfare

**DOI:** 10.1177/00236772251396319

**Published:** 2026-03-09

**Authors:** Jordi L Tremoleda, Aurora Brønstad, Heidrun Potschka, Petra Seebeck, Oliver Stiedl, Vootele Voikar, Sara Wells, TEATIME Animal Welfare Subgroup

**Affiliations:** 1 Blizard Institute, Queen Mary University London, UK; 2 Department of Clinical Medicine, University of Bergen, Norway; 3 Institute of Pharmacology, Toxicology, and Pharmacy, Ludwig-Maximilians-Universität, Munich, Germany; 4 Zurich Integrative Rodent Physiology, University of Zurich, Switzerland; 5 Vrije Universiteit Amsterdam & Amsterdam UMC, The Netherlands; 6 Helsinki Institute of Life Science, Laboratory Animal Centre, University of Helsinki, Finland; 7 Mary Lyon Centre at MRC Harwell, Harwell Science and Innovation Campus, Oxford, UK

**Keywords:** Behaviour, refinement, ethics and welfare, welfare assessments, humane endpoints, home cage systems

## Abstract

COST Action TEATIME unites experts to advance automated monitoring technologies for laboratory animals, with a focus on Home Cage Monitoring (HCM) systems. The use of HCM has great potential to revolutionise welfare monitoring by enabling continuous, non-invasive tracking of physiological and behavioural patterns in group-housed animals within their undisturbed housing environment. These systems capture spontaneous behaviours – such as feeding, grooming, social interactions and sleep cycles – across day and night phases, offering objective data for welfare and scientific assessments. This real-time monitoring might allow for early detection of distress, disease progression and subtle welfare changes, supporting timely interventions and refined humane endpoints. Unlike traditional clinical scoring, which relies on brief daily observations, HCM provides longitudinal, individualised insights and reduces observer bias. It might also facilitate better characterisation of positive affective states, contributing to more holistic welfare evaluations. Despite technological progress, challenges remain in data integration, sensitivity and standardisation across facilities. Effective implementation requires real-time alert capabilities, robust data management, and interdisciplinary collaboration among scientists, veterinarians and data experts. HCM systems should complement – not replace – human expertise, enriching welfare monitoring and scientific reproducibility. Their integration can improve husbandry, refine severity assessments, advancing both animal welfare and scientific replicability.

## Résumé

COST action TEATIME réunit des experts pour faire progresser les technologies de surveillance automatisée des animaux de laboratoire, en mettant l’accent sur les systèmes de surveillance en cage (HCM). L’utilisation des HCM présente le grand potentiel de pouvoir révolutionner la surveillance du bien-être en permettant un suivi continu et non invasif des modèles physiologiques et comportementaux chez les animaux hébergés en groupe dans leur environnement de logement non perturbé. Ces systèmes capturent les comportements spontanés, tels que l’alimentation, le toilettage, les interactions sociales et les cycles de sommeil, à travers les phases de jour et de nuit, offrant des données objectives pour évaluer le bien-être et les paramètres scientifiques. Cette surveillance en temps réel peut permettre une détection précoce de la détresse, de la progression de la maladie et des changements subtils du bien-être, soutenant ainsi des interventions opportunes et des critères d’évaluation plus précis. Contrairement à la notation clinique traditionnelle, qui repose sur de brèves observations quotidiennes, les systèmes HCM fournissent des informations longitudinales et individualisées et réduisent le biais de l’observateur. Ils peuvent également faciliter une meilleure caractérisation des états affectifs positifs, contribuant ainsi à des évaluations plus holistiques du bien-être. Malgré les progrès technologiques, des défis subsistent en matière d’intégration, de sensibilité et de normalisation des données dans l’ensemble des installations. Une mise en œuvre efficace nécessite des capacités d’alerte en temps réel, une gestion robuste des données et une collaboration interdisciplinaire entre scientifiques, vétérinaires et experts en données. Les systèmes HCM devraient compléter, et non remplacer, l’expertise humaine, enrichir la surveillance du bien-être et la reproductibilité scientifique. Leur intégration peut améliorer l’élevage, affiner les évaluations de gravité, faire progresser à la fois le bien-être animal et la reproductibilité scientifique.

## Herausforderungen und Erwartungen hinsichtlich des Einsatzes automatisierter Heimkäfig-Überwachungssysteme zur Verbesserung der Pflege und des Wohlergehens von Labortieren

COST Action TEATIME vereint Experten, um automatisierte Überwachungstechniken für Labortiere zu fördern, wobei der Schwerpunkt auf Home Cage Monitoring (HCM)-Systemen liegt. Der Einsatz von HCM hat großes Potenzial, die Überwachung des Tierwohls zu revolutionieren, da es eine kontinuierliche, nicht-invasive Verfolgung der physiologischen und Verhaltensmuster von in Gruppen gehaltenen Tieren in ihrer ungestörten Umgebung ermöglicht. Diese Systeme erfassen spontane Verhaltensweisen – wie Fressen, Fellpflege, soziale Interaktionen und Schlafzyklen – über Tag und Nacht hinweg und liefern objektive Daten für die Bewertung des Wohlergehens und für wissenschaftliche Untersuchungen. Diese Echtzeitüberwachung kann die frühzeitige Erkennung von Stress, Krankheitsverläufen und subtilen Veränderungen des Wohlergehens ermöglichen und so zeitnahe Interventionen und verfeinerte humane Endpunkte unterstützen. Im Gegensatz zum traditionellen klinischen Scoring, das sich auf kurze tägliche Beobachtungen stützt, liefert HCM langfristige, individualisierte Erkenntnisse und reduziert die Beobachterverzerrung. Es kann auch eine bessere Charakterisierung positiver affektiver Zustände bieten und so zu einer ganzheitlicheren Bewertung des Wohlbefindens beitragen. Trotz des technologischen Fortschritts bestehen weiterhin Schwierigkeiten hinsichtlich der Integration, Sensibilität und Standardisierung von Daten zwischen Einrichtungen. Eine effektive Umsetzung erfordert Echtzeit-Warnfunktionen, ein robustes Datenmanagement und eine interdisziplinäre Zusammenarbeit zwischen Wissenschaftlern, Tierärzten und Datenexperten. HCM-Systeme sollten menschliches Fachwissen ergänzen – nicht ersetzen – und so die Überwachung des Wohlergehens und die wissenschaftliche Reproduzierbarkeit verbessern. Ihre Integration kann die Tierhaltung verbessern, die Bewertung des Schweregrads verfeinern und sowohl das Tierwohl als auch die wissenschaftliche Replizierbarkeit fördern.

## Desafíos y expectativas en el uso de la monitorización automatizada en jaula de alojamiento para avanzar en el cuidado y bienestar de los animales de laboratorio

Resumen

La Acción COST TEATIME reúne a expertos para impulsar tecnologías de monitorización automatizada aplicadas a animales de laboratorio, con un enfoque en los sistemas de monitorización en jaula de alojamiento (Home Cage Monitoring, HCM). El uso de HCM tiene un gran potencial para revolucionar la monitorización del bienestar, al permitir el seguimiento continuo y no invasivo de patrones fisiológicos y conductuales en animales alojados en grupo dentro de su entorno habitual, sin perturbaciones. Estos sistemas registran comportamientos espontáneos —como alimentación, acicalamiento, interacciones sociales y ciclos de sueño— tanto en fases diurnas como nocturnas, arrojando datos objetivos para la evaluación del bienestar y de parámetros científicos. La monitorización en tiempo real puede permitir la detección temprana de signos de malestar, progresión de enfermedad y cambios sutiles en el bienestar, facilitando intervenciones oportunas y el refinamiento de criterios humanitarios de finalización. A diferencia de la puntuación clínica tradicional, que se basa en observaciones breves diarias, HCM ofrece información longitudinal y personalizada, reduciendo el sesgo del observador. También puede facilitar una mejor caracterización de estados afectivos positivos, contribuyendo a evaluaciones del bienestar más holísticas. A pesar de los avances tecnológicos, persisten desafíos en la integración de datos, sensibilidad y estandarización entre instalaciones. Su implementación efectiva requiere capacidades de alerta en tiempo real, una gestión de datos sólida y colaboración interdisciplinar entre científicos, veterinarios y especialistas en datos. Los sistemas HCM deben complementar —y no sustituir— la experiencia humana, enriqueciendo la monitorización del bienestar y la reproducibilidad científica. Su integración puede mejorar las prácticas de manejo y cuidado animal, refinar la evaluación de la severidad y contribuir al avance tanto del bienestar animal como de la reproducibilidad científica.

Since 2021 the COST Action TEATIME^1^ has brought together animal research professionals from across Europe to promote the development of emerging technologies that support automated welfare monitoring for laboratory animals. Notably, advances in Home Cage Monitoring (HCM) systems enable continuous 24/7 assessment of various physiological and behavioural patterns in group-housed animals in their home cages.^2^ These technologies facilitate the automatic collection of spontaneous, non-stimulated behaviours in natural housing conditions, including feeding and drinking, social interactions and activity levels, all within a safe, enriched and non-experimentally challenged environment. Upcoming technological developments provide promise for new add-on sensor systems and analysis modules for HCM. Thereby, the impact on the 3Rs is significant, offering objective and quantifiable welfare and scientific assessments across both day and night phases over extended periods.^3^

The greatest advantage of HCM systems lies in their ability to monitor animals non-invasively and continuously, detecting early, subtle or sporadic indicators of disease progression or welfare concerns – signals often missed during routine out-of-cage behavioural testing or brief daily observations, especially in nocturnal species observed during daylight, working hours. Depending on the system used, multiple parameters can be measured directly, such as activity levels, food and water intake, scratching, well-being sensitive behaviours such as grooming, burrowing or nest building in mice, social interactions, abnormal (e.g. stereotypic or fighting) behaviour and locomotor impairments, and physiological data (e.g. surface or body temperature, heart rate, respiratory rate). Behavioural metrics such as circadian rhythms and sleep patterns can also be captured.^4,5^ This real-time in vivo monitoring is particularly relevant for improving husbandry practices, but also characterisation of genetically modified animals (e.g. AppNL-G-F Alzheimer’s disease mouse model;^6^ or N171-82Q mouse model of Huntington’s disease^7^) and severity assessment (e.g. early detection of motor impairment,^7,8^ sleep-related disturbances^9^ to improve care monitoring) and assessing responses to therapeutic interventions.^10^ These capabilities enhance the sensitivity and temporal accuracy of identifying adverse effects and their progression, enabling early intervention and informed decisions regarding experimental and/or humane endpoints.

Despite a growing body of literature on laboratory animal care and welfare monitoring,^11–13^ most protocols still rely on punctual observations of standardised clinical parameters (by filling in the clinical score sheets) – typically including body weight/condition, posture, coat condition, mobility, response to stimuli, and system-specific indicators such as respiration, mucosal colour, faeces, urine or grimace scales. Such parameters, while critically relevant, are generally assessed through daily observations and cumulative scoring systems, which might offer limited discrimination and longitudinal tracking of individual metrics. Scoring approaches might also use evoked behaviour responses (e.g. nociceptive tests), which can confoundingly also trigger stress. HCM systems have reported progressive changes in locomotion patterns on lung cancer mouse models following 24/7 daily serial monitoring prior to changes on standard measures such as body weight drop, allowing for an earlier identification of tumour onset and better discrimination for the implementation of supporting care and/or progressing towards advanced monitoring such as tumour imaging.^8^ Similarly, reduced nocturnal distance travelled and climbing activity as disease symptoms were detected in preclinical models of Huntington’s disease, prior to the any overt clinical signs of the disease onset.^7,14^ The advantage of observing the mice undisturbed within their home cage and social groups over multiple light/dark cycles across serial days/weeks allows to disentangle specific temporal alterations that could be missed through time limited daily checks. Such discriminatory and prolonged temporal assessment is particularly critical for conditions such as Huntington’s disease, which has a slow development of early cognitive symptoms followed by a critical worsening of movements and coordination functions. These examples exemplify the role of HCM systems to support earlier identification of disease-driven phenotypic changes, thus allowing for better tailored care programmes.

HCM systems, supported with digitised video-tracking, offer the potential to automate and continuously monitor multiple behavioural measures, reducing operator bias, supporting remote monitoring and enabling reliable digital alerts to promptly identify animals in pain or distress. This facilitates timely implementation of necessary corrective actions and supports the detection of time-sensitive changes during active/dark phases, allowing for more comprehensive behavioural and physiological assessments. HCM can be successfully implemented to optimise pain management following surgical interventions, looking at relevant welfare traits such as nesting and burrowing.^15^ Similarly, HCM recorded activities such as voluntary wheel running have been reported as a more sensitive indicator of cumulative impact of repeated intraperitoneal injections on pancreatic cancer murine models than clinical scoring.^16^ Such a digital biomarker has also proven predictive of age-dependent changes to support severity assessments and identifiable changes nearing endpoint in aged mice.^17^

Automated alert data can complement standard clinical observations, supporting prolonged monitoring data to strengthen severity assessments tailored to the cumulative effects on individual animals throughout experimental procedures. Such complementary data are instrumental in refining welfare monitoring programmes across the lifespan of the study, supporting composite measures for severity assessment and improving the detection of predictive signs of humane endpoint.

## Challenges and actions to facilitate acceptance and use

HCM equipment ranges from sophisticated, commercially available cages with integrated monitoring and data processing software to more affordable systems assembled in-house using components such as video cameras, and microphones for ultrasonic vocalisation.^18^

The capabilities of sensors and cameras to report on physiological and behavioural states are rapidly evolving, with increasingly sensitive detection systems being developed. However, the adoption of such multimodal and multivariate methods (e.g. digitised animal tracked data and video recordings) within the laboratory animal community, in particular, those responsible for overseeing animal care and welfare, requires training to provide methodological awareness and, critically, data management support. The expectations for the HCM to improve and to harmonise severity assessment protocols, allowing for better comparability of protocols across animal models, users, and facilities, is a multidisciplinary community endeavour. Working groups with expertise on animal welfare, researchers, HCM technologist and data experts must work together to better define the physiology–pathology status transition in view of contributing variables such as age, sex, genotype and environmental conditions. This is particularly relevant by reviewing longitudinal data and agreeing on the welfare relevance of newly assessed behaviour or physiological data patterns.

A key requirement for effective welfare monitoring is that HCM systems operate in real time. To be impactful, systems must detect deviations from normal behavioural or physiological ranges and issue timely digital alerts. Additionally, the ability to integrate and compare multiple parameters across individuals and monitoring during across different timelines is essential for tracking disease progression and evaluating treatment efficacy. Challenges remain, such as limited sensitivity to detect subtle changes (e.g. grimacing) or sporadic, short-lived events (e.g. seizures), especially in group-housed animals with similar appearance for which individual ID tagging may be required. Nonetheless, the acquisition of such datasets offers a unique opportunity to identify more precise and applicable biomarkers to determine, for example, disease progression in animal models for better determination of experimental and/or humane endpoints. Such data should be easily accessible to the animal welfare staff as specific critical variables such as body temperature, weight and activity, or, as agreed by expert groups, as a composite index validated for a specific experimental model. Engagement across users, laboratories and institutions to use of HCM systems would build up larger data resources, enabling better guidance on specific models and experimental settings.^19^

For the laboratory animal community to embrace these rapidly developing technologies, clear analytical and technological support must be integrated within species-specific experimental settings and biosafety constraints. Systems must be versatile, adaptable to evolving experimental needs, support timely alerts and be logistically feasible and cost-effective. Data storage and processing pose additional challenges and highlight the need to facilitate logistical capabilities for data sharing (with large amounts of stored data) and supporting expertise for analysis.^20^ To support data accessibility and timely analysis, experimental units must engage with collaborative data sharing initiatives, aiming to promote open-source analysis software to foster openness and engagement. Scalability and cross-disciplinary collaboration among data scientists, biologists, veterinarians and animal welfare experts will be crucial.

When adopting digital alert-based welfare approaches, it is essential to incorporate phenotyping data from exemplary animal care practices across laboratories. Skilled behavioural specialists, providing generally acceptable (unambiguous/objective) operational definitions for specific behaviours, must collaborate with data scientists and computational experts during coding and analysis. Cross-disciplinary partnerships involving data scientists, machine learning engineers, biologists, veterinarians and animal welfare specialists are vital to develop relevant skillsets for HCM innovation. HCM data must be accessible and interpretable to support metadata generation and broader uptake. One promising approach is the creation of a central repository for HCM data, enabling method development and reference datasets for use by researchers, technologists and data integrators.^20^

## Conclusions

HCM systems represent a transformative advancement in laboratory animal welfare monitoring, as valuable complementary tools that enhance traditional welfare evaluations by enabling more comprehensive, time-sensitive and individualised animal monitoring. This will facilitate earlier identification of adverse events and promote timely care and supportive interventions and identifiable experimental and/or humane endpoints, ultimately advancing both animal welfare and scientific quality as a major contribution to refinement.

As technological capabilities continue to evolve, it is essential that the laboratory animal community actively participates in the implementation and critical evaluation of HCM-based datasets. Collaborative engagement across scientists, veterinarians and animal welfare professionals with data scientists will be key to unlocking the full potential of these systems. Importantly, the integration of HCM systems must be accompanied by continued recognition of the importance of direct, in-person welfare assessments and is the shared responsibility of all animal users to uphold the highest standards of daily care.
